# Case study of GBMT post-bariatric surgery with psychiatric co-morbidities

**DOI:** 10.1192/j.eurpsy.2025.1408

**Published:** 2025-08-26

**Authors:** S. Ramos-Perdigués, Ï. Robert-Montaner, M. Martín-Fernández, M. Pérez, J. Ramos, O. Sarroca, C. Mur de Viu

**Affiliations:** 1 Psychiatry, Hospital Nostra Senyora de Meritxell, Escaldes-Engordany, Andorra; 2 Psychiatry, Gestió de Serveis Sanitaris, La Seu d’Urgell, Spain; 3 Sherwood Forest Hospitals NHS Foundation Trust, Sutton in Ashfield, United Kingdom; 4 Internal Medicine; 5 Psychology, Hospital Nostra Senyora de Meritxell, Escaldes-Engordany, Andorra

## Abstract

**Introduction:**

Gelatinous bone marrow transformation (GBMT) is a degenerative change in hematopoietic bone marrow, initially linked to anorexia nervosa (AN) and recently to bariatric surgery (BS) (Böhm *et al*. Am J Surg Pathol 2000; 24(1) 56-65). BS is associated to malabsorption, nutrient deficits, diets, and medical complications resembling eating disorders (ED) (Conceição *et al*. Int J Eat Disord 2023; 56(4) 831-834), complicating diagnosis and management. Treating the underlying cause is key for GBMT recovery.

**Objectives:**

Review BS complications: malnutrition, GBMT and AN with normal Body Mass Index (BMI).

**Methods:**

This case study highlight the emergence of GBMT and atypical AN following BS.

**Results:**

A 55-year-old woman first sought treatment from the Community Mental Health Team (CMHT) over 30 years ago for marital issues and was given medication. In 2006, she underwent bariatric surgery (BS) due to morbid obesity (BMI 52.6 kg/m²). Since 2007, she had 3 hospitalizations for cosmetic surgeries and 5 for medical complications, including oedema, hypoproteinaemia, pancytopenia, zinc deficiency, and sepsis requiring ICU admission. She also showed symptoms of depressive disorder (DD), AN, and purgative symptoms. In 2017, she was re-referred to CMHT and diagnosed with DD, anorexia was considered a symptom of DD.

In 04/2024 she was readmitted under Internal Medicine care with subacute multifactorial diarrhoea, severe malnutrition, pancytopenia, coagulopathy, vitamins A, E, D and Zn deficiency and lower limb oedema (likely contributing to a normal BMI).

Diarrhoea was managed by switching sertraline to citalopram and budesonide added to treat lymphocytic colitis. Moreover, ciprofloxacin and metronidazole were used for small intestinal bacterial overgrowth.

Psychiatric involvement confirmed DD, anxiety disorder (AD), and atypical AN. Topiramate, mirtazapine and olanzapine were stopped to possible myelotoxic effects. A bone marrow aspirate confirmed GBMT. Benzodiazepines and gabapentin were used to manage AD. Her malnutrition was managed with Total Parenteral Nutrition and she was transferred to a psychiatric ward where she received specific treatment for ED. Pancreatic enzymes were added to reduce malabsorptive impact of BS. The option of reversing the bypass was considered.

Once the vital risk decreased and she was consuming normalized intakes, she was discharged with a BMI=20.9.

One month later, she was euthymic, reduced anxiety and coagulopathy and nutritional parameters normalized with haematological improvement.

**Conclusions:**

Given the high prevalence of malnutrition and ED post-BS, ED should be systematically assessed even in patients with normal BMI. Early diagnosis prevents worsening of symptoms, which only improve after nutritional recovery (Steinhausen *et al.* Am J Psychiatry 2002; 159(8) 1284-93). Multidisciplinary management is crucial to achieve optimal nutritional outcomes.

**Disclosure of Interest:**

None Declared

